# Supplementary material for: Niobium-doped layered cathode material for high-power and low-temperature sodium-ion batteries
Source: Nat Commun. 2022 Jun 9;13:3205. doi: 10.1038/s41467-022-30942-z (PMC9184510; doi:10.1038/s41467-022-30942-z)
Supplement: Supplementary file 1 — Supplementary Information [file 41467_2022_30942_MOESM1_ESM.pdf]

Supplementary Information

Niobium-doped layered cathode material for high-power and low-temperature sodium-ion batteries

Qinhao Shi[1], Ruijuan Qi[2], Xiaochen Feng[1], Jing Wang[3], Yong Li[1], Zhenpeng Yao[4], Xuan Wang[1], Qianqian Li[1], Xionggang Lu[5], Jiujun Zhang[1,6], Yufeng Zhao[1*]

[1] Institute for Sustainable Energy/College of Sciences, Shanghai University, Shanghai 200444, P. R. China. [2] Key Laboratory of Polar Materials and Devices (MOE) and Department of Electronics, East China Normal University, Shanghai 200062, P. R. China. [3] Key Laboratory of Applied Chemistry in Hebei Province, Yanshan University, Qinhuangdao, 066004, P. R. China. [4] Center of Hydrogen Science, Shanghai Jiao Tong University, 800 Dongchuan Road, Shanghai 200240, P. R. China. [5] State Key Laboratory of Advanced Special Steel & Shanghai Key Laboratory of Advanced Ferrometallurgy & School of Materials Science and Engineering, Shanghai University, Shanghai 200444, P. R. China. [6] College of Materials Science and Engineering, Fuzhou University, Fuzhou, 350108, China.

Supplementary Figures

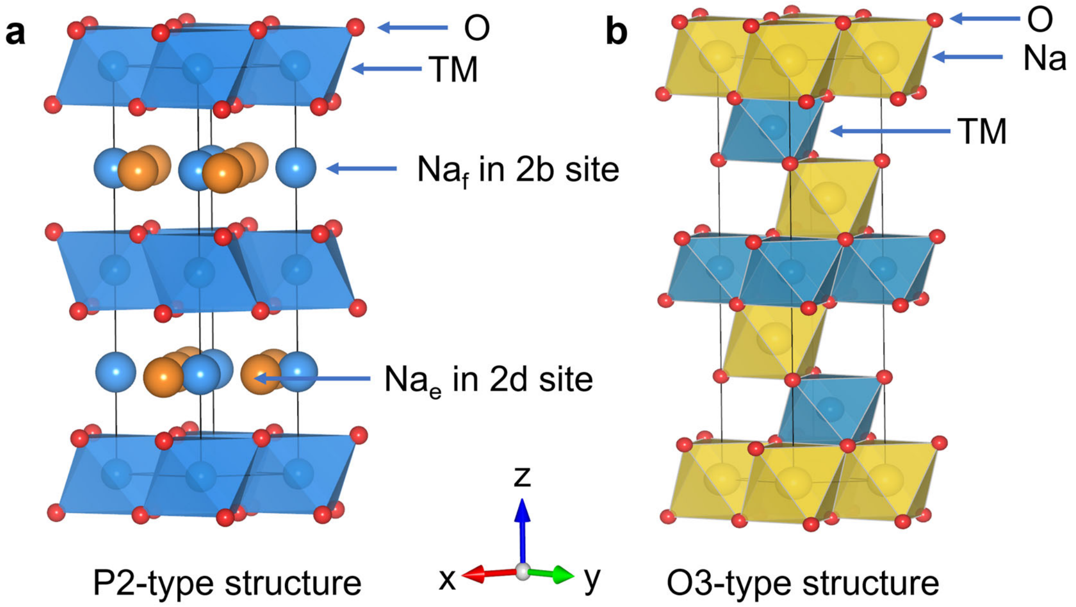

Supplementary Figure. 1 The P2-and O3-type crystal structures. a The hexagonal structure of P2-type oxide with the space group of *P63/mmc* (194). b The Rhombohedral structure O3-type oxide with the space group of *R-3m* (166). The TM stands for transition metal.

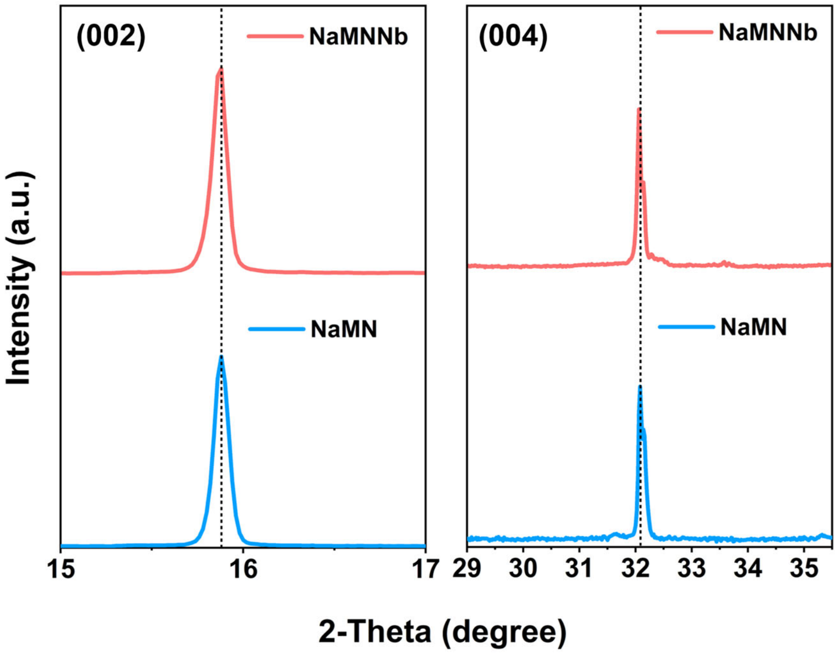

Supplementary Figure. 2 Shift of the (002) and (004) diffraction peak of P2-NaMN and P2-NaMNNb compounds.

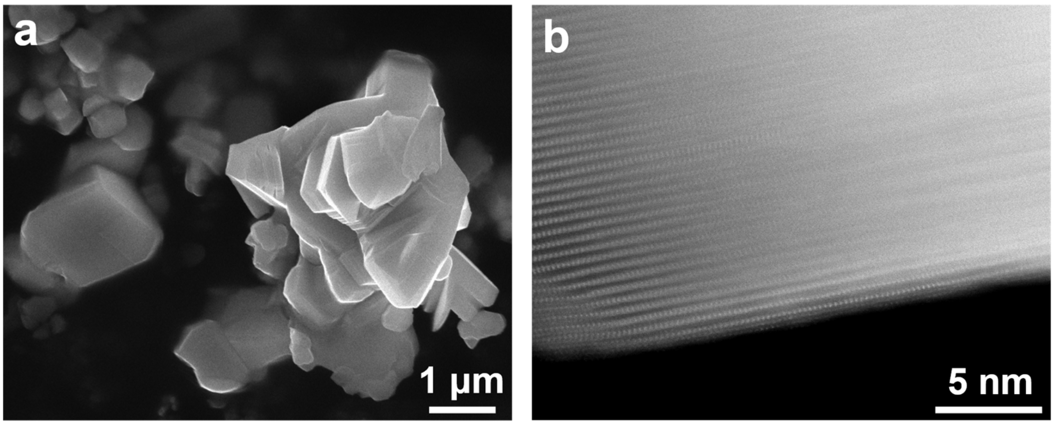

Supplementary Figure. 3 a Field-emission scanning electron microscope (SEM) image of P2-NaMNNb compound. b HADDF-STEM image that illustrates the surface reconstruction layer of P2-NaMNNb only exists along [100] direction.

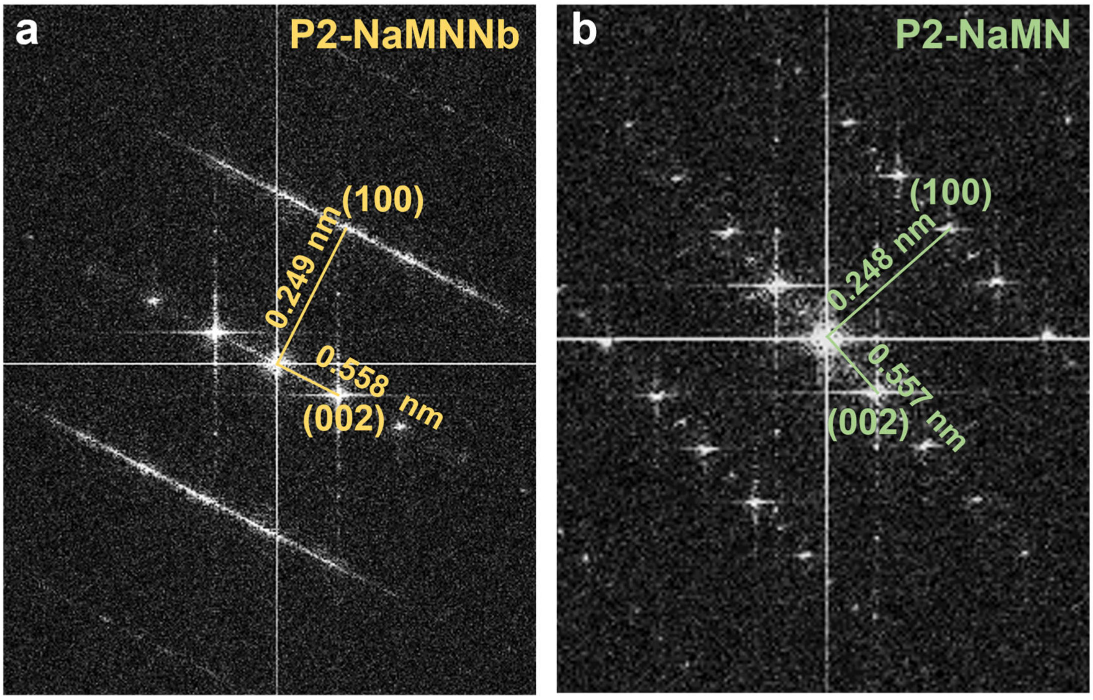

Supplementary Figure. 4 Fast Fourier Transform (FFT) diffraction of a, P2-NaMNNb and b, P2-NaMN.

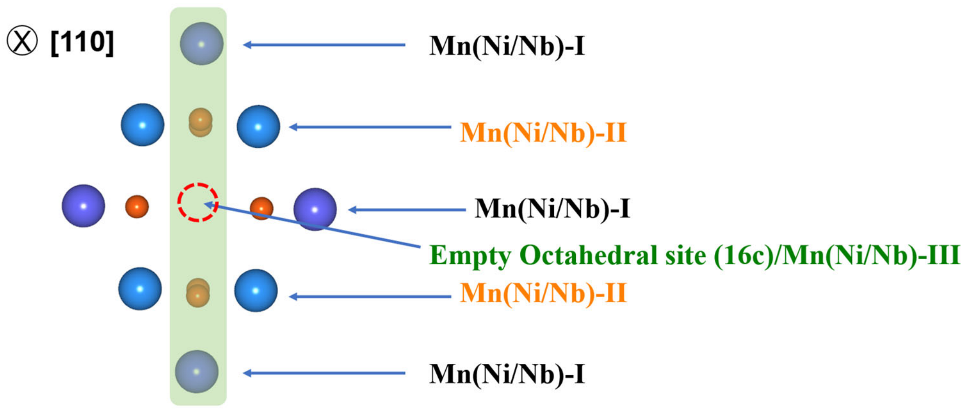

Supplementary Figure. 5 Schematic diagram of spinel-like-atomic structure projected along [110] crystallographic direction.

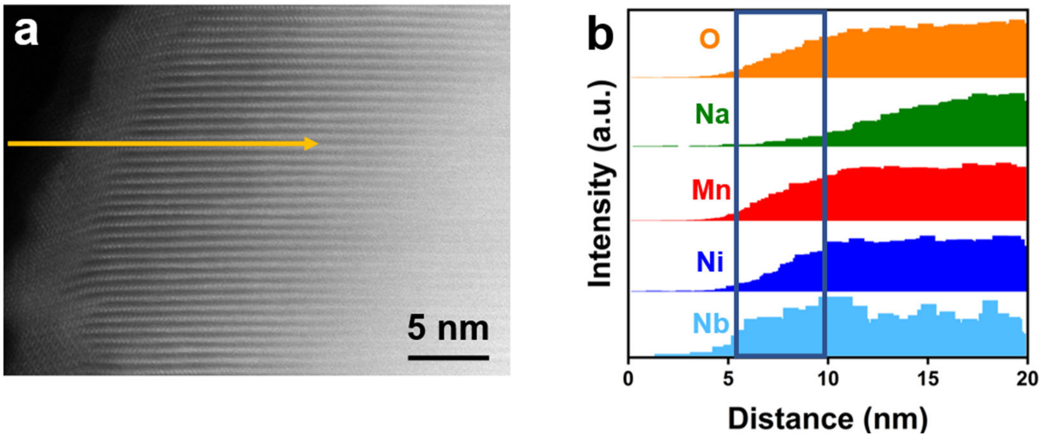

Supplementary Figure. 6 a STEM-HAADF image showing Energy Spectrum Line Scan position. b Corresponding Energy Spectrum elemental line profiles of a, O (orange), Na (green), Mn (red), Ni (blue) and Nb (cyan-blue).

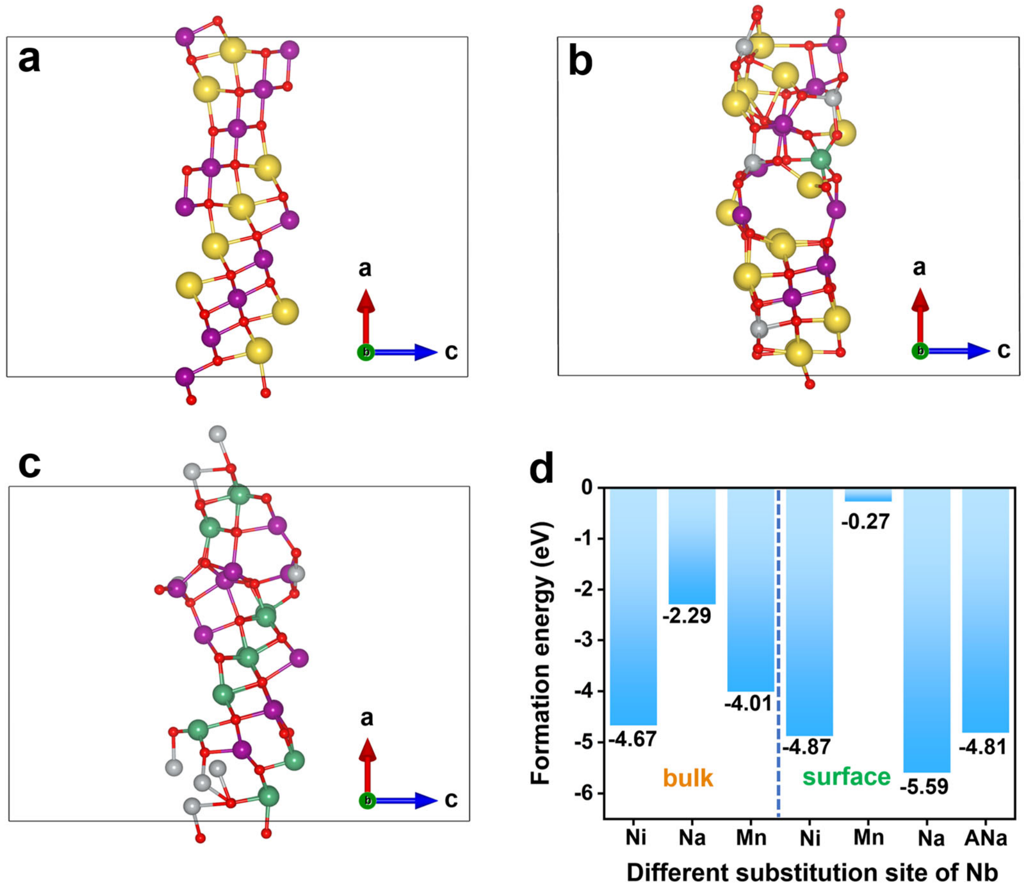

Supplementary Figure. 7 Schematic diagram of the substitution of Nb^{5+} in bulk and surface of (-103) plane of P2-NaMNNb. a The original plane, b Replace single Na^{+}, c Replace all Na^{+}. d The comparison of formation energy of different substitution site of Nb (ANa represent all Na are replaced by Nb in the surface layer). See detailed description in Supplementary Note 1.

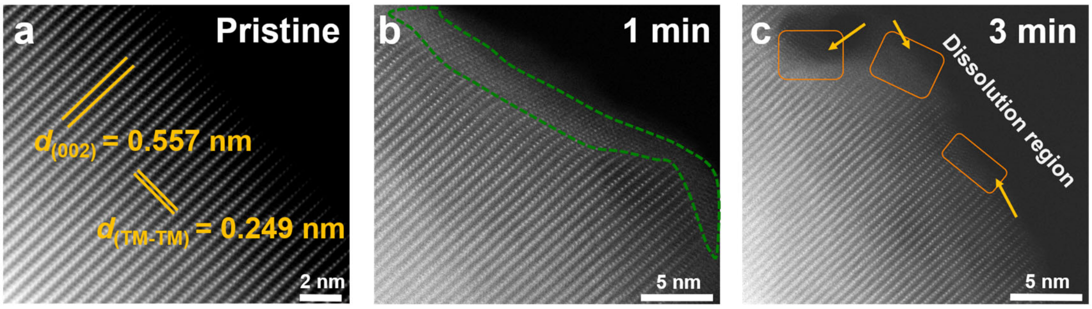

Supplementary Figure. 8 a-c, HAADF images of the $Na_{0.78}Ni_{0.32}Mn_{0.68}O_2$ particles projected along [010] zone axes taken after different electron beam irradiation times (0 min, 1 min and 3 min) with a dose rate of ~1.12×10^4 electrons $Å^{-2}$ s^{-1}, at a dwell time of ~1μs/pixel.

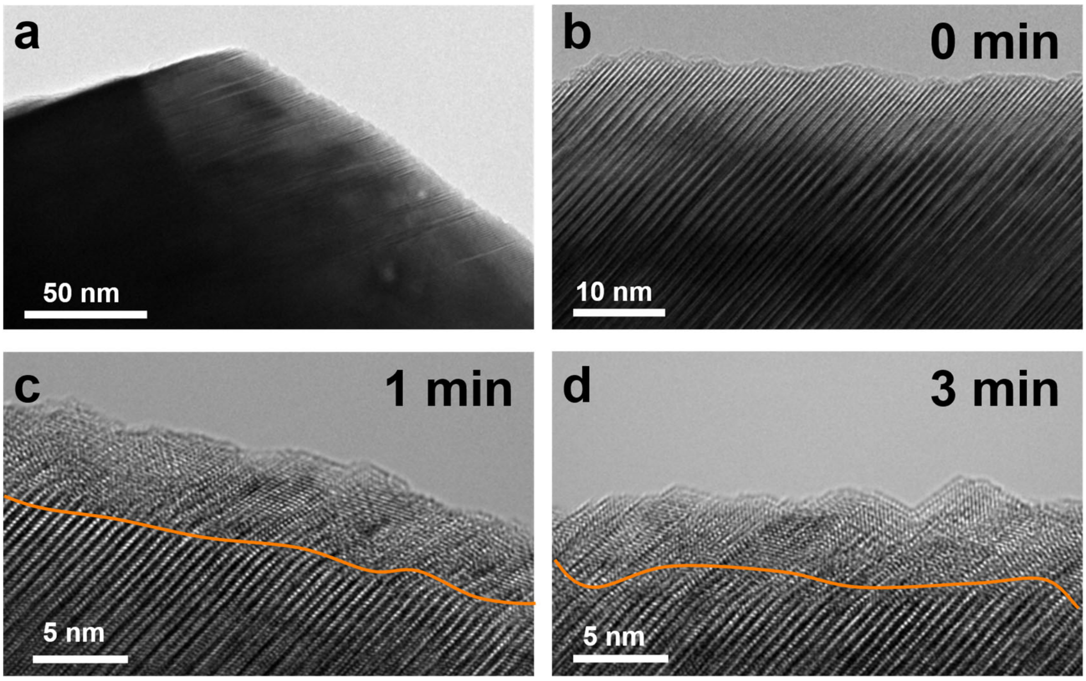

Supplementary Figure. 9 a, HRTEM image of the $Na_{0.78}Ni_{0.32}Mn_{0.68}O_2$ projected along [010] zone axis, and taken after different electron beam irradiation times (0 min, 1 min and 3 min corresponding to b, c, d.

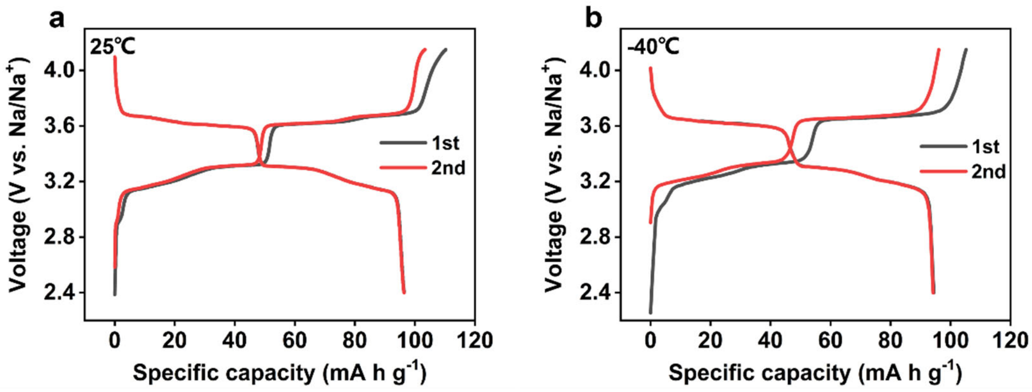

Supplementary Figure. 10. The first two charge-discharge curves of Na||P2-NaMNNb coin cell in the voltage range of 2.4-4.15 V at 92 mA g^{-1} a, 25 °C and b, -40 °C.

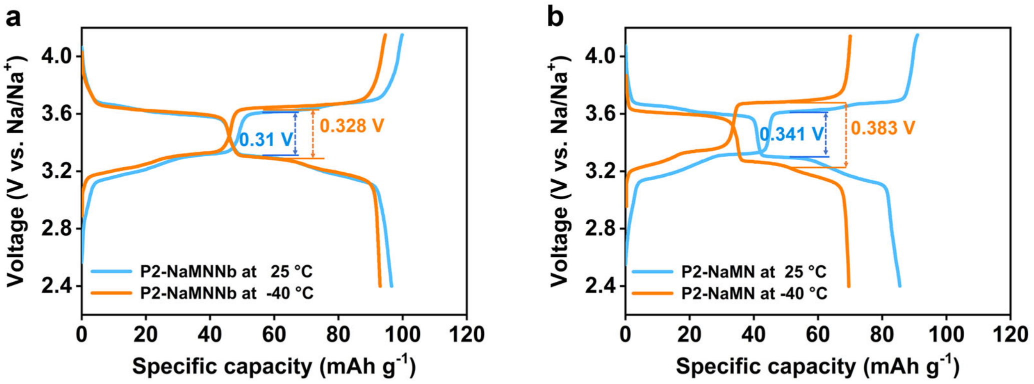

Supplementary Figure. 11 The charge–discharge curves of a, P2-NaMNNb and b, P2-NaMN cathode in the voltage range of 2.4-4.15 V in coin cell at 25 °C and -40 °C; 92 mA g^{-1}.

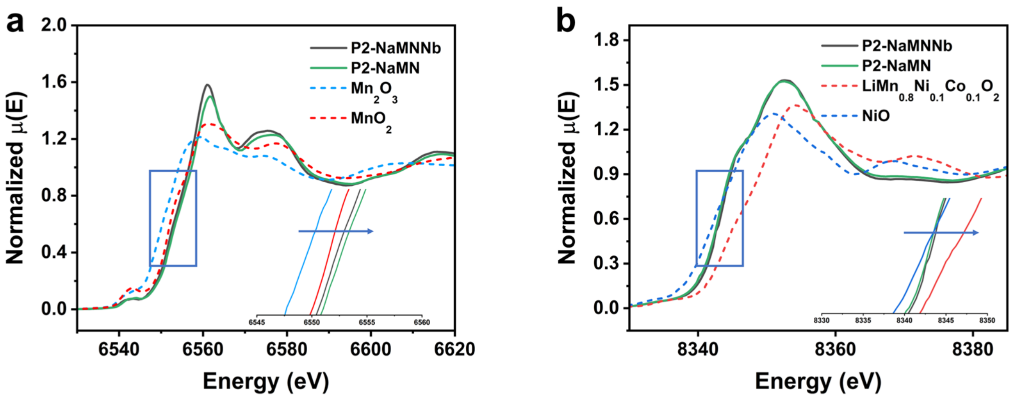

Supplementary Figure. 12 The X-ray absorption near edge structure (XANES) of a, Mn and b, Ni K-edge of P2-NaMNNb and P2-NaMN as well as standard metal oxide references. See detailed analysis in Supplementary Note 2.

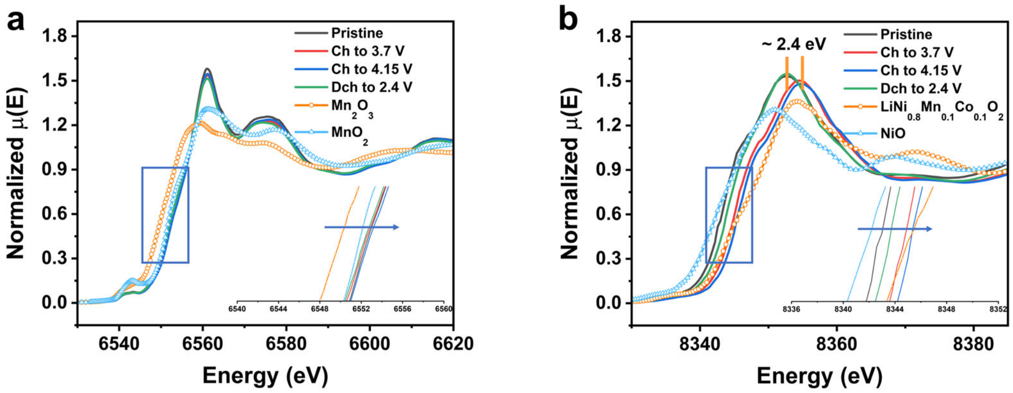

Supplementary Figure. 13 The ex situ XANES of P2-NaMNNb with different cutoff voltage a, Mn; b, Ni. See detailed analysis in Supplementary Note 3.

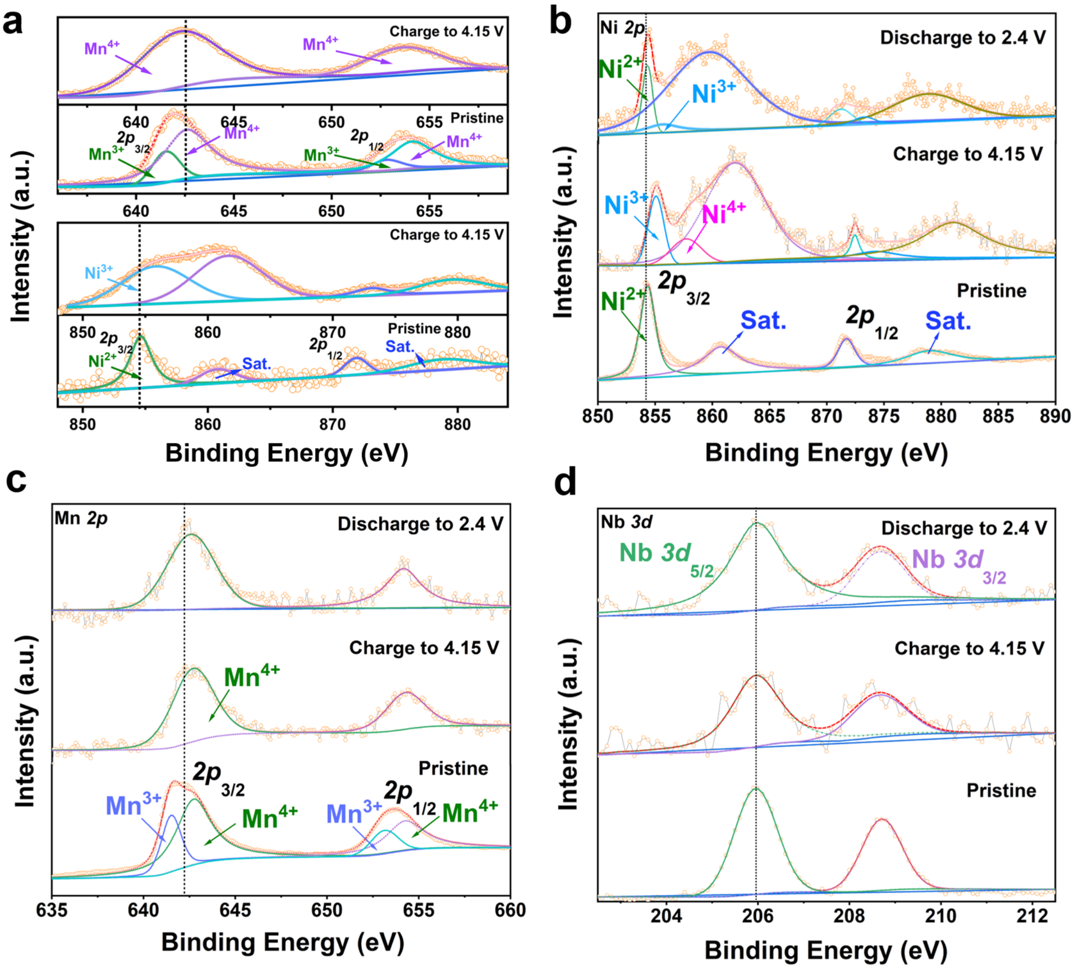

Supplementary Figure. 14 The ex situ XPS analysis of a Ni 2*p* and Mn 2*p* for P2-NaMN at pristine state, charge to 4.15 V. b, Ni 2*p*; c, Mn 2*p* and d, Nb 3*d* for P2-NaMNNb at pristine state, charge to 4.15 V and discharge to 2.4 V, respectively. See detailed analysis in Supplementary Note 4

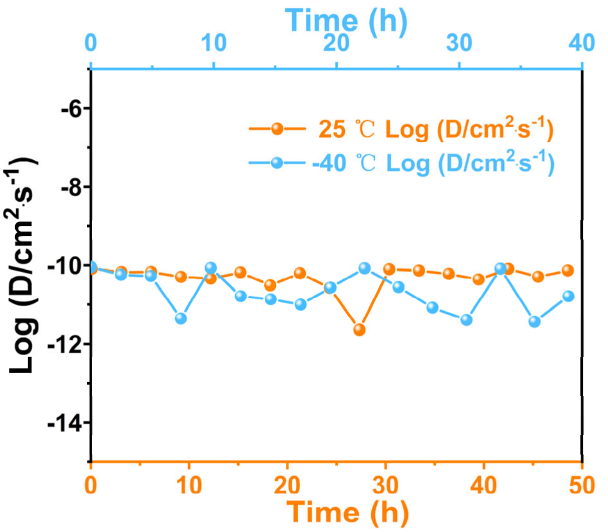

Supplementary Figure. 15 Calculated Na^+ diffusion coefficients of Na||P2-NaMN coin cell from GITT at 25 and -40 °C.

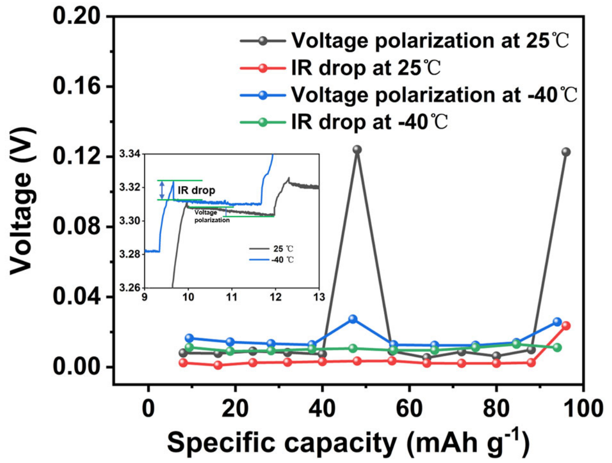

Supplementary Figure. 16 Calculated voltage polarization and IR-drop from GITT data of Na||P2-NaMNNb coin cell during charge process. See detailed analysis in Supplementary Note 5.

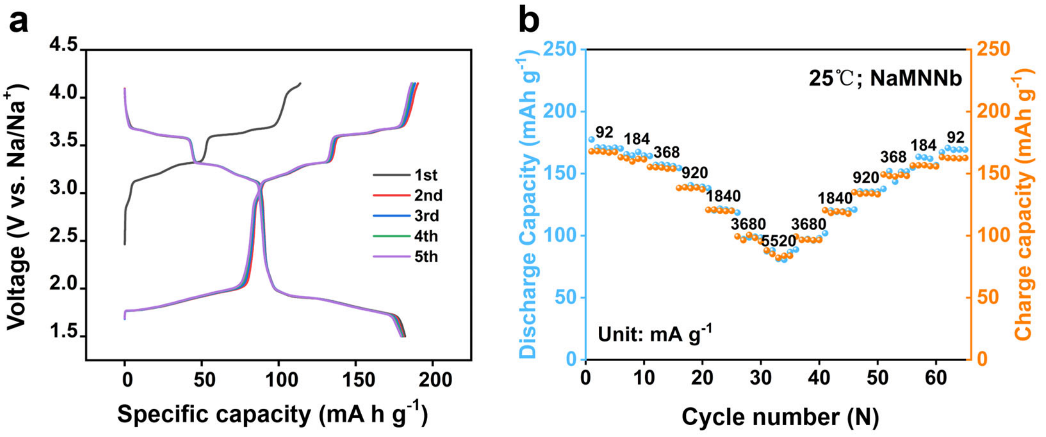

Supplementary Figure. 17 a Charge/discharge curves of Na||P2-NaMNNb coin cell at 92 mA g^{-1}, 25 °C in 1.5-4.15 V. b The corresponding rate performance at 25 °C.

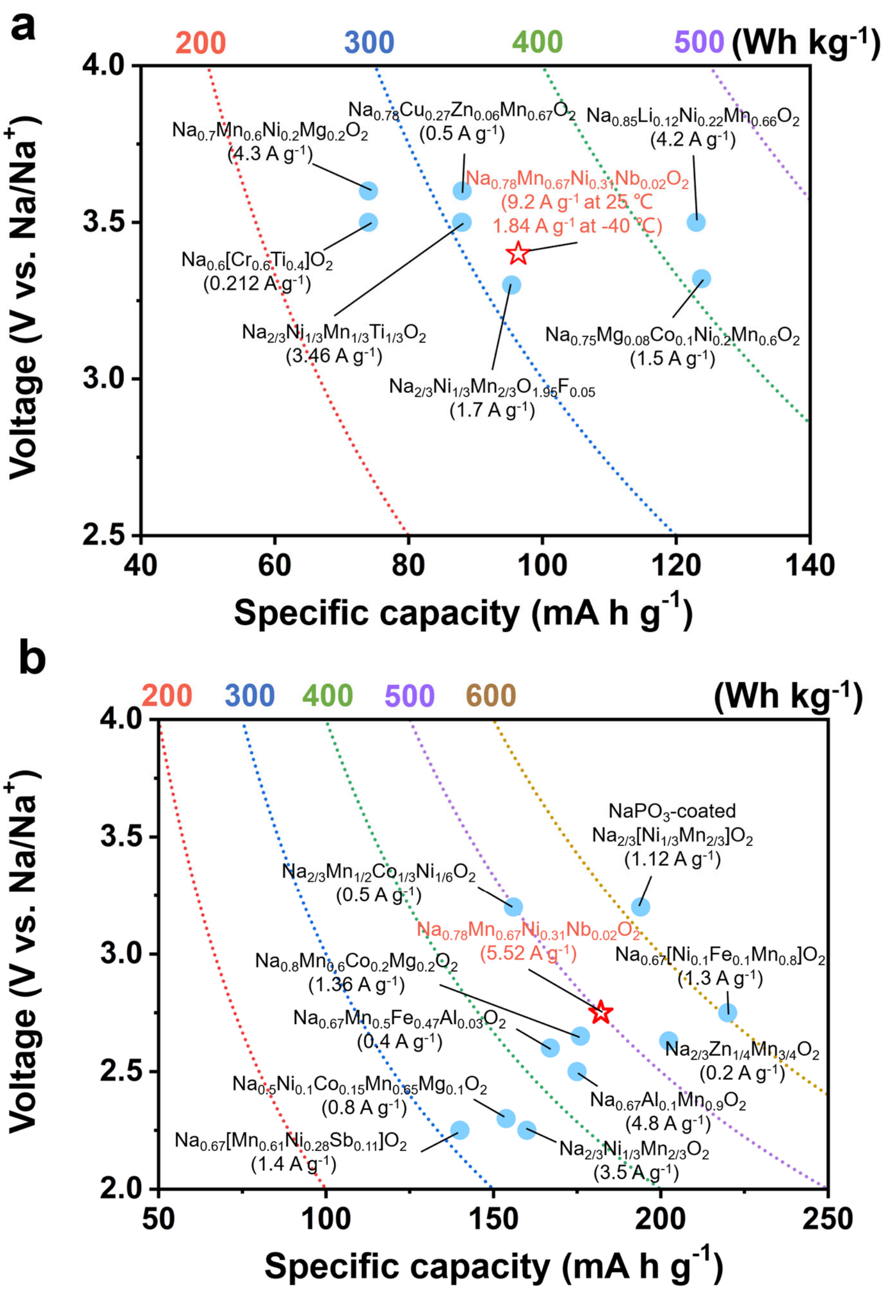

Supplementary Figure. 18 Comparison of half-cell performance with reported cathode materials for SIBs in coin cells. Plots of specific capacity versus operating voltage with calculated specific energy, as well as the maximum specific current curves, a, in narrow voltage range ((ΔV= cut-off voltage at charge state minus cut-off voltage at discharge state) > 2.0 V or Mn ion participate the redox reaction.); b, in wide voltage range (ΔV < 2.0 V or Mn ion participate the redox reaction). The data are consistent with those in Supplementary Table 5.

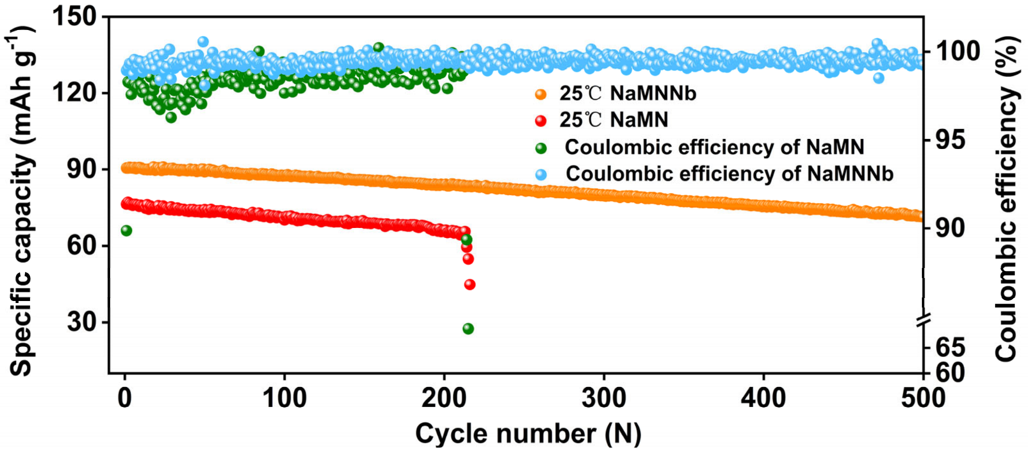

Supplementary Figure. 19 Long-term cycling stability of Na||P2-NaMNNb coin cell at specific current of 920 mA g^{-1} at 25 °C between 2.4-4.15 V.

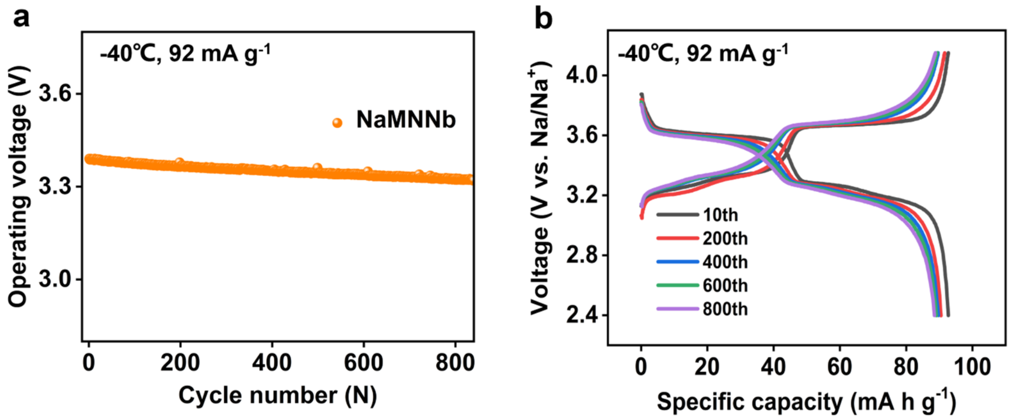

Supplementary Figure. 20 a The average working voltage (> 3.3 V) at specific current of 92 mA g^{-1} at -40 °C of Na||P2-NaMNNb coin cell in the voltage range of 2.4-4.15 V. b Corresponding charging and discharging curves of various cycle numbers at -40 °C at specific current of 92 mA g^{-1}.

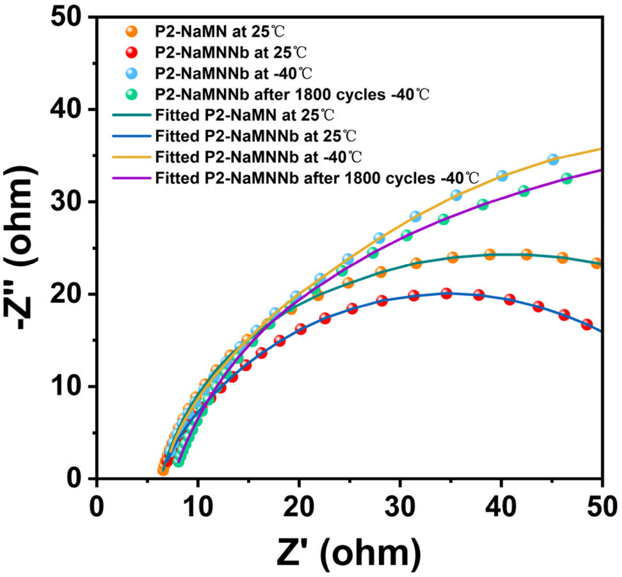

Supplementary Figure. 21 Magnified EIS curves of P2-NaMNNb and P2-NaMN at 25 °C and -40 °C.

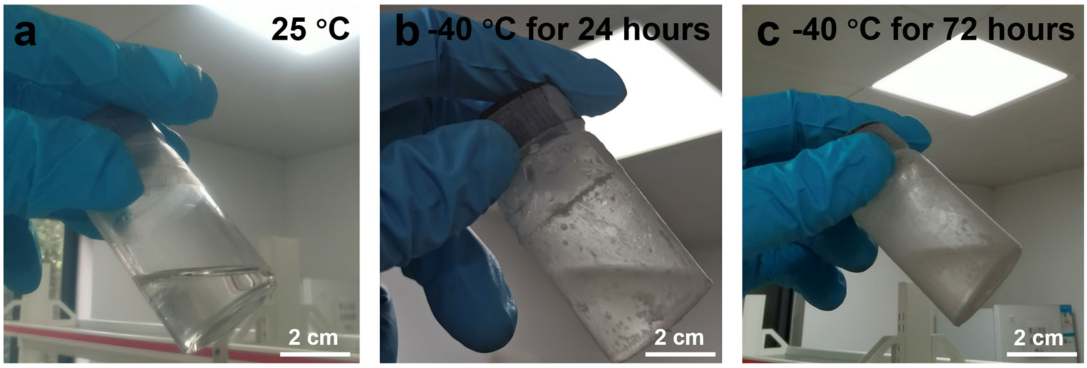

Supplementary Figure. 22 Photographic pictures of the glass vials containing the non-aqueous electrolyte solution at 25 °C (a) and at -40 °C for (b) 24 hours and (c) 72 hours.

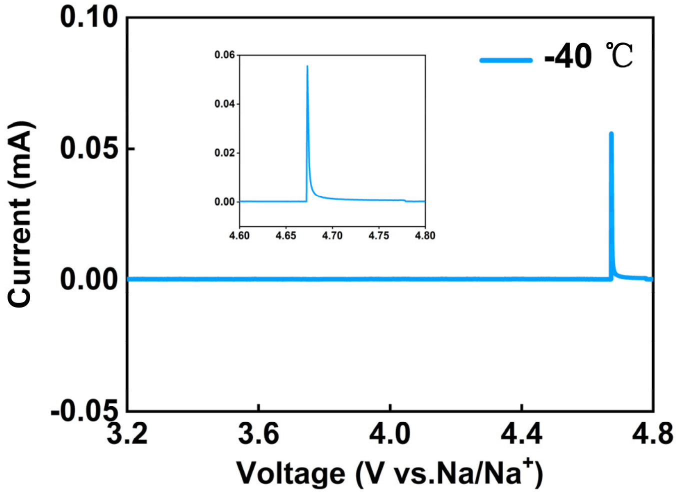

Supplementary Figure. 23 LSV curves of 1 mol $NaPF_6$ in Diglyme at 0.2 mV s^{-1} based on Na||Al coin cells at -40 °C between 3.2-4.8 V.

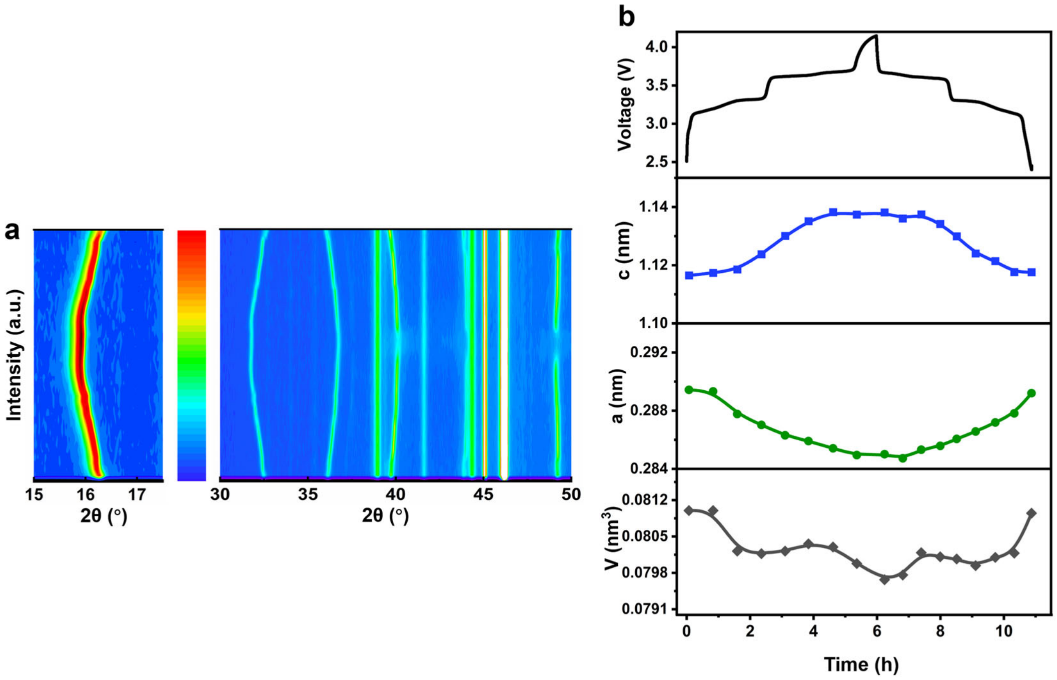

Supplementary Figure. 24 Structure evolution of P2-NaMNNb upon Na^+ extraction/insertion. a The typical intensity contour maps of in situ XRD patterns corresponding to the charge and discharge curves at 20 mA g^{-1} between 2.4-4.15 V of Swagelok cell at 25 °C. b The variation of cell parameters *a/b* (green lines), *c* (blue lines), and *V* (gray lines). P.S No characteristic peak of P2-NaMNNb in in situ XRD patterns was observed from 18 to 30 2*Theta* during the charge and discharge process, so the signals of this section are not included.

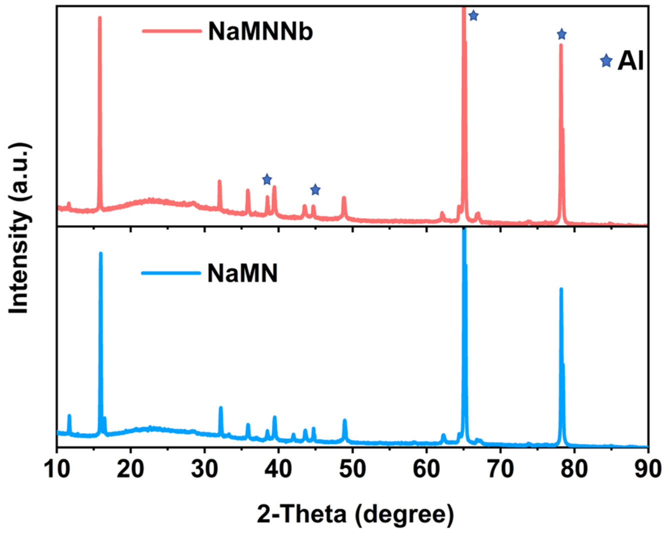

Supplementary Figure. 25 Comparison of X-ray diffraction patterns of P2-NaMNNb and P2-NaMN at 92 mA g^{-1} in coin cell after 30 cycles at 25 °C. The electrodes after disassembling process were washed with diglyme for three times and dried in an argon-filled glove box, then the surface of electrode were protected with polyimide film from 3M company.

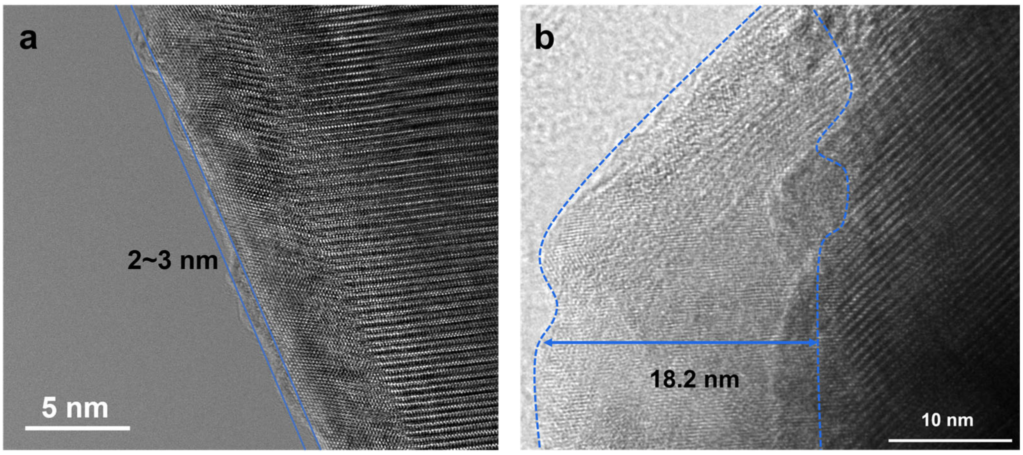

Supplementary Figure. 26 The HRTEM image of a, P2-NaMNNb and b, P2-NaMN at 92 mA g^{-1} in coin cell after 30 cycles at 25 °C. The electrodes after disassembled process were used diglyme to wash for one time and dried in an argon-filled glove box.

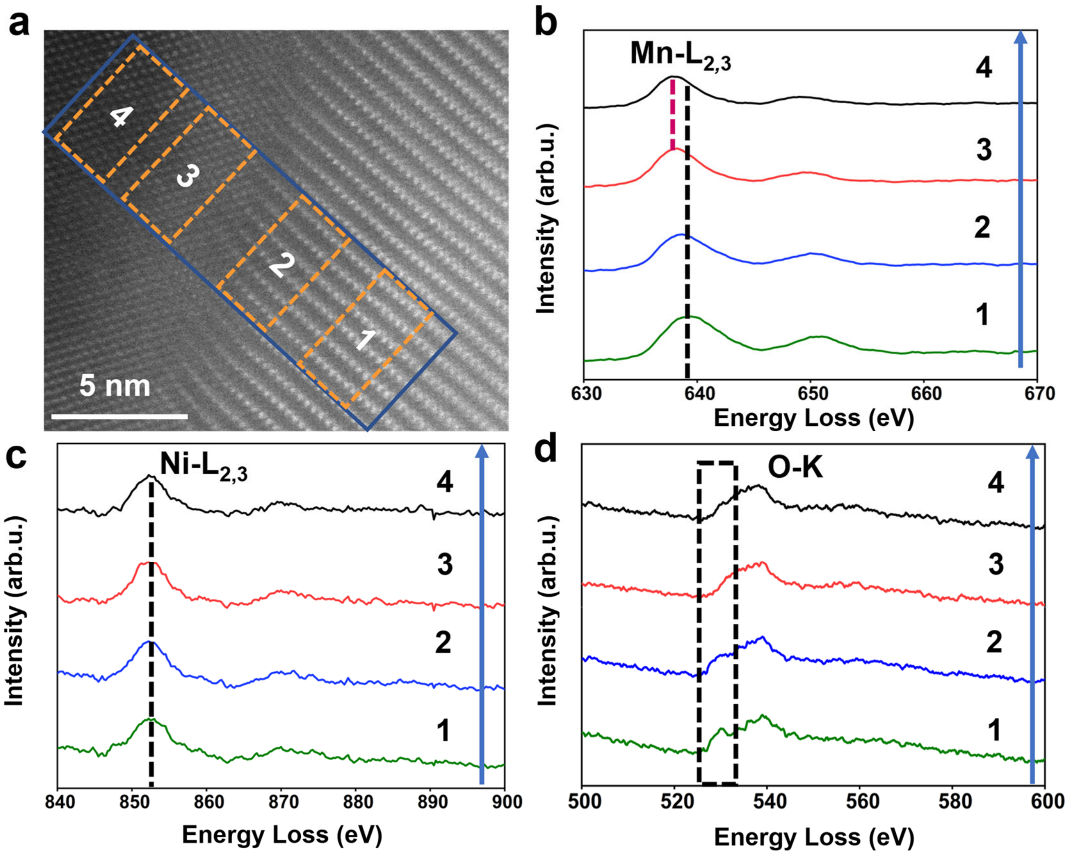

Supplementary Figure. 27 The image of EELS area of P2-NaMNNb powder a the HADDF-STEM and EELS spectra of b, Mn L-edge; c, Ni L-edge; d, O K-edge. See detailed analysis in Supplementary Note 6.

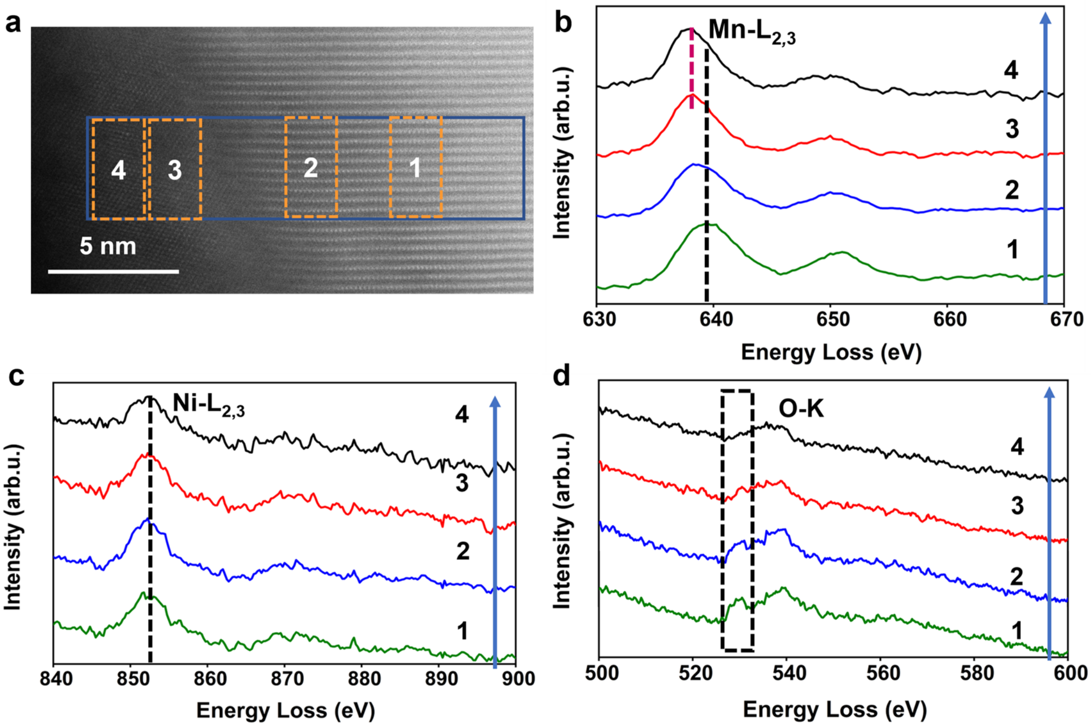

Supplementary Figure. 28 a The image of EELS area of P2-NaMNNb, which stopped at 2.4 V at 92 mA g^{-1} in coin cell in discharge state after 30 cycles at 25 °C. The EELS spectra of b, Mn L-edge; c, Ni L-edge; d, O K-edge. The electrodes after disassembled process were used diglyme to wash for three time and dried in an argon-filled glove box.

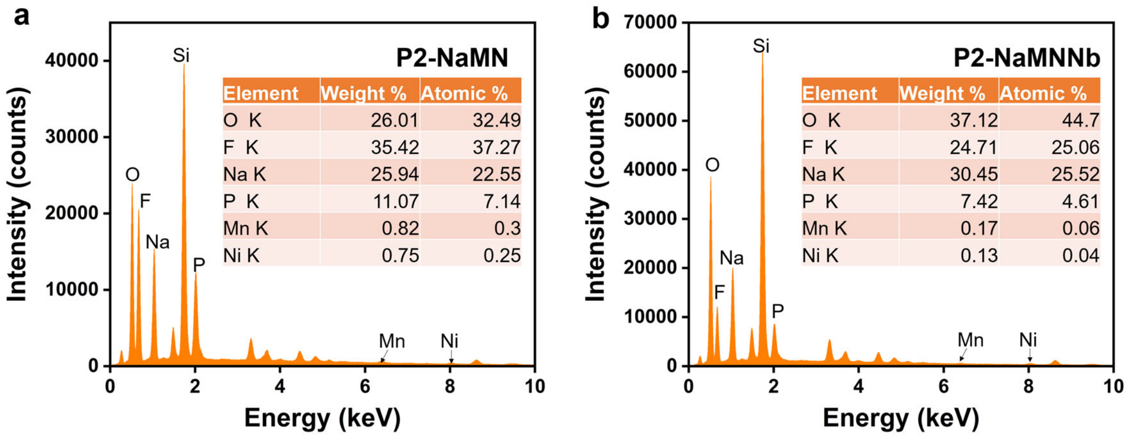

Supplementary Figure. 29 Energy Dispersive X-ray spectroscopy (EDX) spectra of a P2-NaMN, b P2-NaMNNb of Na anodes, which stopped at 2.4 V at 92 mA g^{-1} in coin cell in discharge state after 30 cycles at 25 °C.

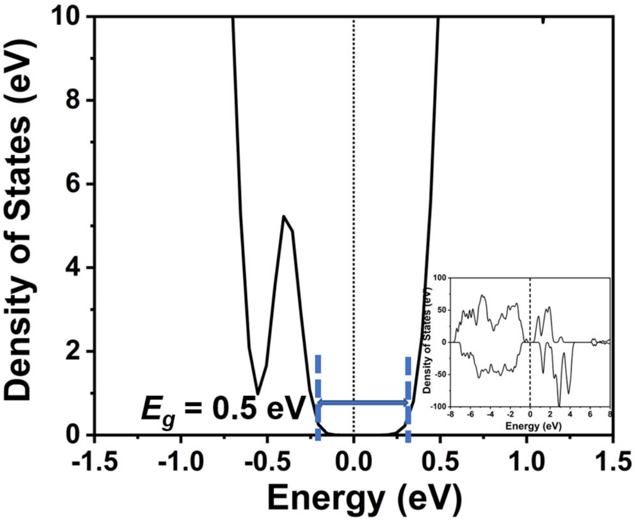

Supplementary Figure. 30 The total density of state of $Na_{24}Ni_{10}Mn_{22}O_{64}$.

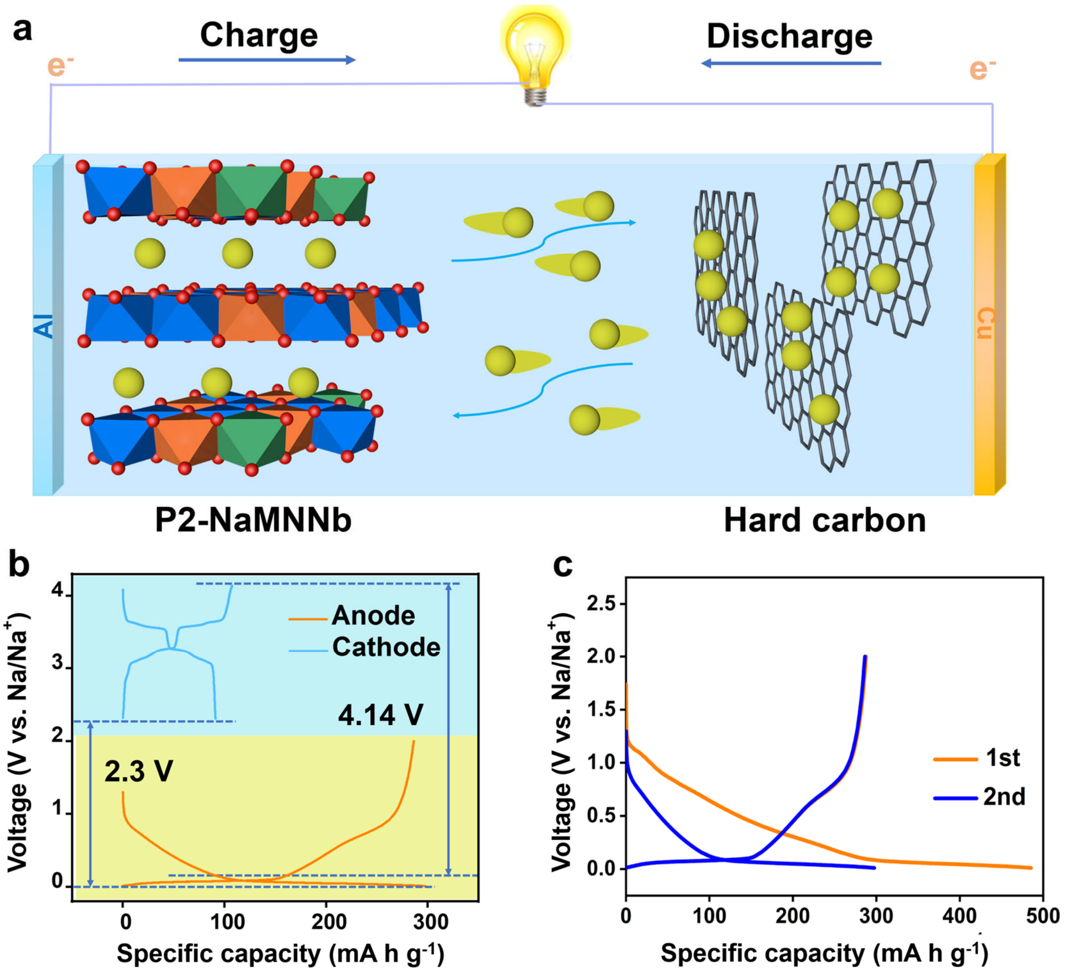

Supplementary Figure. 31 a Schematic of pre-sodiated hard carbon||P2-NaMNNb coin cell. b Potential profiles versus specific capacity of P2-NaMNNb (2.4-4.15 V) and hard carbon electrodes (0.01-2 V). c Potential profiles of the hard carbon electrode in Na metal coin cell configuration at 25 °C between 0.01-2.0 V at 50 mA g^{-1}.

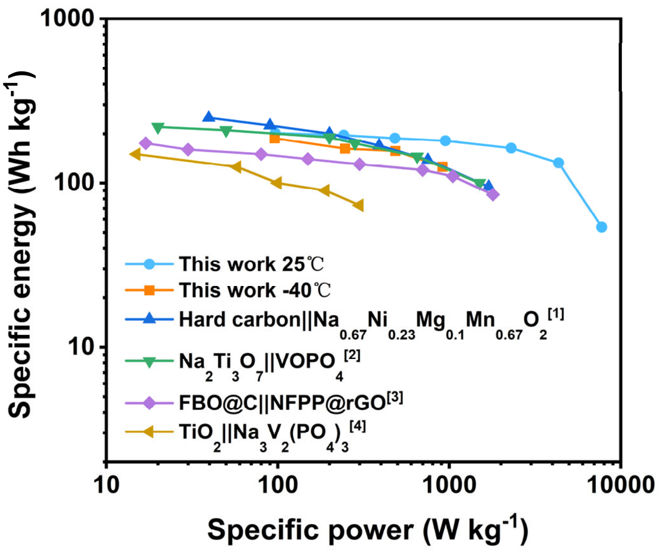

Supplementary Figure. 32 The comparison of the specific power and specific energy for the present research work with other Na-ion lab-scale cells reported in the literature[1-4]

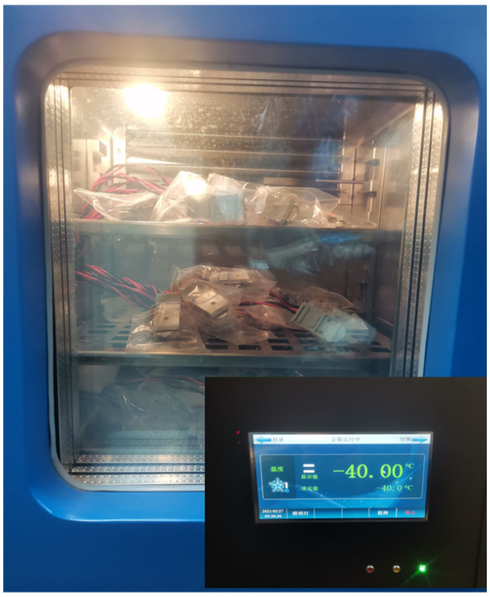

Supplementary Figure. 33 The image of the low temperature electrochemical test for half-cell and full-cell at -40 °C.

Supplementary Tables

Supplementary Table 1. Stoichiometry from the inductively coupled plasma atomic emission spectrometry (ICP-AES) analysis. The results presented as stoichiometric values, which are calculated from the mass fraction of each element.

ICP results (mol mL^{-1})	Na	Mn	Ni	Nb
$Na_{0.78}Mn_{0.67}Ni_{0.31}Nb_{0.02}O_2$	0.78	0.67	0.31	0.02

Supplementary Table 2. Crystallographic parameters of P2-NaMNNb refined by the Rietveld method.

site	*x*	*y*	*z*	*Occ.*
Na_f	0	0	1/4	0.237
Na_e	2/3	1/3	3/4	0.543
Mn	0	0	0	0.670
Ni	0	0	0	0.310
Nb	0	0	0	0.02
O	2/3	1/3	0.07520	1
a = 0.2888(2) nm	c = 1.1155(4) nm	V = 0.08056(2) nm^3	R_p = 2.36%	R_{wp} = 4.19%

Supplementary Table 3. Crystallographic parameters of P2-NaMN refined by the Rietveld method.

site	*x*	*y*	*z*	*Occ.*
Na_f	0	0	1/4	0.303
Na_e	2/3	1/3	3/4	0.477
Mn	0	0	0	0.68
Ni	0	0	0	0.32
O	2/3	1/3	0.07239	1
a = 0.2887(1) nm	c = 1.1148(2) nm	V = 0.08046(1) nm^3	R_p = 3.17%	R_{wp} = 5.61%

Supplementary Table 4. The formation energy of the different substitution site of Nb.

Formation energy (eV)			
original ion	Ni	Na	Mn
bulk	-4.67	-4.01	-2.29
surface	-4.88	-5.59	-0.27

Supplementary Table 5. The comparison of electrochemical performance of various cathode oxide material in coin cell. The negative electrode is Na disc and the operating temperature is about 25-30 °C.

	Positive electrode/mass loading (mg cm^{-2}) and active material content (wt.%)	Average cell discharge voltage/potential window (V)	Specific capacity/specific current (mAh g^{-1}/mA g^{-1})	Rate performance and corresponding specific current (mAh g^{-1} / A g^{-1})	Electrolyte	Ref
Wide-voltage range	$Na_{0.67}Al_{0.1}Mn_{0.9}O_2$ (Not given / 80)	2.5 (2-4)	175/12	74 (4.8)	1 mol L^{-1} $NaClO_4$ in PC:FEC=98:2, vol%	5
	$Na_{2/3}Ni_{1/3}Mn_{2/3}O_2$ (2.5 / 75)	2.25 (1.5-4.0)	160/17	73.4 (3.5)	1 mol L^{-1} $NaClO_4$ in PC:FEC=95:5, vol%	6
	$NaPO_3$-coated $Na_{2/3}[Ni_{1/3}Mn_{2/3}]O_2$ (8 / 80)	3.2 (1.5-4.3)	194/20	115 (1.12)	0.5 mol L^{-1} $NaPF_6$ in PC:FEC, 98:2, vol%	7
	$Na_{0.67}Mn_{0.5}Fe_{0.47}Al_{0.03}O_2$ (Not given / 75)	2.6 (1.5-4.2)	167/10	67 (0.4)	1 mol L^{-1} $NaClO_4$ in PC/ EC, 50/50 vol%	8
	$Na_{0.5}Ni_{0.1}Co_{0.15}Mn_{0.65}Mg_{0.1}O_2$ (2-3 / 70)	2.3 (1.5-4.0)	153.8/16	97.5 (0.8)	1 mol L^{-1} $NaClO_4$ in PC:FEC=98:2, vol%	9
	$Na_{0.67}[Mn_{0.61}Ni_{0.28}Sb_{0.11}]O_2$ (2 / 70)	2.25 (1.8-4.2)	140/14	82 (1.4)	1 mol L^{-1} $NaClO_4$ in PC/ EC, 50/50, and 5% FEC vol%	10
	$Na_{2/3}Zn_{1/4}Mn_{3/4}O_2$ (1.4 / 70)	2.63 (1.5-4.5)	202.4/20	140 (0.2)	Not given	11
	$Na_{0.67}[Ni_{0.1}Fe_{0.1}Mn_{0.8}]O_2$ (4 / 85)	2.75 (1.5-4.3)	220/26	120 (1.3)	0.5 mol L^{-1} $NaPF_6$ in PC:FEC, 98:2, vol%	12
	$Na_{2/3}Mn_{1/2}Co_{1/3}Ni_{1/6}O_2$ (1.5-2 / 80)	3.2 (1.5-4.5)	~190/20	70 (0.5)	1 mol L^{-1} $NaClO_4$ in PC/ EC, 50/50 vol%	13
	$Na_{0.8}Mn_{0.6}Co_{0.2}Mg_{0.2}O_2$ (2.75 / 70)	2.65 (1.6-4.4)	176/17	68.8 (1.36)	1 mol L^{-1} $NaClO_4$ in PC/ EC, 50/50, and 5% FEC vol%	14
	$Na_{0.78}Mn_{0.67}Ni_{0.31}Nb_{0.02}O_2$ (2-3 / 80)	2.75 (1.5-4.15)	182.1/92	88.8 (5.52)	1 mol L^{-1} $NaPF_6$ in diglyme	This work
Narrow-voltage range	$Na_{0.78}Cu_{0.27}Zn_{0.06}Mn_{0.67}O_2$ (1.5 / 80)	3.6 (2.5-4.1)	88/10	73 (0.5)	1 mol L^{-1} $NaClO_4$ in PC/ EC, 50/50 vol%	15
	$Na_{0.7}Mn_{0.6}Ni_{0.2}Mg_{0.2}O_2$ (2.0 / 70)	3.6 (2.5-4.2)	74/34	60 (4.3)	1 mol L^{-1} $NaClO_4$ in PC/ EC, 50/50 vol%	16
	$Na_{2/3}Ni_{1/6}Mn_{2/3}Cu_{1/9}Mg_{1/18}O_2$ (2.0 / 70)	3.5 (2.5-4.15)	87.9/60	64.0 (3.6)	1 mol L^{-1} $NaClO_4$ in PC:FEC=95:5, vol%	17
	$Na_{2/3}Ni_{1/3}Mn_{2/3}O_{1.95}F_{0.05}$ (2-3.5 / 80)	3.3 (2-4)	95.4/17	86.4 (1.7)	1 mol L^{-1} $NaClO_4$ in PC:FEC=95:5, vol%	18
	$Na_{0.85}Li_{0.12}Ni_{0.22}Mn_{0.66}O_2$ (3-4 / 80)	3.5 (2-4.3)	123/22.4	79.3 (4.2)	1 mol L^{-1} $NaClO_4$ in PC/ EC, 50/50, and 5% FEC vol%	19
	$Na_{0.75}Mg_{0.08}Co_{0.1}Ni_{0.2}Mn_{0.6}O_2$ (2 / 75)	3.32 (2-4.3)	123.8/30	79.8 (1.5)	1 mol L^{-1} $NaPF_6$ in PC:FEC=95:5, vol%	20
	$Na_{0.6}[Cr_{0.6}Ti_{0.4}]O_2$ (Not given / 75)	3.5 2.5-3.85	74/10.6	61 (0.212)	1 mol L^{-1} $NaClO_4$ or 0.8 mol L^{-1} $NaPF_6$ in EC/DEC 4:6 vol%	21
	$Na_{2/3}Ni_{1/3}Mn_{1/3}Ti_{1/3}O_2$ (3-4 / 75)	3.5 (2.5-4.15)	88/17.3	68.2 (3.46)	1 mol L^{-1} $NaPF_6$ in DEC/EC, 50/50, and 5% FEC vol%	22
	$[Na_{0.67}Zn_{0.05}]Ni_{0.18}Cu_{0.1}Mn_{0.67}O_2$ (1.5-1.8 / 70)	3.6 (2.5-4.35)	103/17	38 (3.4)	1 mol L^{-1} $NaClO_4$ in PC:FEC=95:5, vol%	23
	$Na_{0.78}Mn_{0.67}Ni_{0.31}Nb_{0.02}O_2$ (2-3 / 80)	3.4 (2.4-4.15)	96.6/92	65.8 (9.2)	1 mol L^{-1} $NaPF_6$ in diglyme	This work

Supplementary Table 6. The fitted data of EIS measurements.

	P2-NaMN at 25 °C	P2-NaMNNb at 25 °C	P2-NaMNNb at -40 °C	P2-NaMNNb at -40 °C after 1800 cycles
R0 (Ω)	5.781	5.414	4.969	6.458
R1 (Ω)	62.21	58.61	109.1	89.53
Q_1 ($S \cdot S^{\alpha1}$)	0.0000091247	0.000012368	0.000010741	0.000012634
α_1	0.81295	0.74804	0.73407	0.74154
R2 (Ω)	175	123	83.72	113.5
Q_2 ($S \cdot S^{\alpha2}$)	0.00051406	0.00047447	0.00037113	0.00032066
α_2	0.78854	0.88151	0.89084	0.69211
Q_3 ($S \cdot S^{\alpha2}$)	0.0111	0.042836	0.056488	0.026404
α_3	0.54392	0.63436	0.5	0.5
χ^2	0.00049311	0.00032466	0.00029406	0.00047234

R0: Ohmic resistance

R1: the solid electrolyte interphase (SEI) resistance

R2: the charge transfer resistance

Q1: Value corresponding to the admittance ($1/|Z_{Qf}|$) at $\omega = 1.0$ rad/*s* ($S \cdot S^{\alpha1}$)

Q2: Value corresponding to the admittance ($1/|Z_{Qf}|$) at $\omega = 1.0$ rad/*s* ($S \cdot S^{\alpha2}$)

Q3: Value corresponding to the admittance ($1/|Z_{Qf}|$) at $\omega = 1.0$ rad/*s* ($S \cdot S^{\alpha3}$)

α_1: Exponent for CPE1

α_2: Exponent for CPE2

α_3: Exponent for CPE3

χ^2: Chi-squared function about the fitting error for cell.

Supplementary Table 7. The comparison of electrochemical performance of various cathode material in full-cell. The operating temperature is about 25-30 °C.

<table>
<tr><th>Cell configuration and mass loading (mg cm^{-2}) and active material content (wt.%)</th><th>Cell type and voltage range (V)</th><th>Specific energy / Maximum power (based on total active material in cathode and anode) (Wh kg^{-1} / W kg^{-1})</th><th>Initial discharge capacity (based on cathode active material) at the maximum specific current applied (mAh g^{-1}/A g^{-1})</th><th>Electrolyte</th><th>Ref.</th></tr>
<tr><td>Hard carbon (Not given)|| [$Na_{0.67}Zn_{0.05}$]$Ni_{0.18}Cu_{0.1}Mn_{0.67}O_2$ (2 / 70)</td><td>Coin cell 2.4-4.25</td><td>217.9</td><td>~63 (based on total active material in cathode and anode) / 0.85</td><td>1 mol L^{-1} $NaClO_4$ in PC:FEC=95:5, vol%</td><td>[23]</td></tr>
<tr><td>Hard carbon (Not given)|| $Na_{2/3}Ni_{1/3}Mn_{2/3}O_2$ nanofibers (2.5 / 75)</td><td>Coin cell 1.2-3.8</td><td>212.5</td><td>~160 / 1.73</td><td>1 mol L^{-1} $NaClO_4$ in PC:FEC=95:5, vol%</td><td>[6]</td></tr>
<tr><td>Hard carbon (Not given / 80)|| P2/P3-$Na_{0.7}Li_{0.06}Mg_{0.06}Ni_{0.22}Mn_{0.67}O_2$ (Not given / 75)</td><td>Pouch cell 2-4.2</td><td>218</td><td>62.2 / 0.595</td><td>1 mol L^{-1} $NaClO_4$ in PC:FEC=95:5, vol%</td><td>[24]</td></tr>
<tr><td>Hard carbon (Not given)|| $Na_{0.7}Mg_{0.2}[Fe_{0.2}Mn_{0.6}\square_x]O_2$ (Not given / 70)</td><td>Coin cell 1.4-4.4</td><td>165</td><td>121.7 / 0.03</td><td>1 mol L^{-1} $NaClO_4$ in PC/ EC, 50/50, and 5% FEC vol%</td><td>[25]</td></tr>
<tr><td>Hard carbon (Not given/80)|| $Na_{0.76}Ca_{0.05}[Ni_{0.23}\square_{0.08}Mn_{0.69}]O_2$ (2 / 70)</td><td>Pouch cell 1.9-4.2</td><td>257.6</td><td>64 / 2.4</td><td>1 mol L^{-1} NaPF6 in PC, EMC, and 5 vol% FEC.</td><td>[26]</td></tr>
<tr><td>Hard carbon || $Na_{0.67}[Ni_{0.1}Fe_{0.1}Mn_{0.8}]O_2$ (4 / 85)</td><td>Coin cell 1.4-4.2</td><td>542 (based on cathode active material)/3900</td><td>140 / 1.3</td><td>1 mol L^{-1} $NaClO_4$ in PC:FEC=98:2, vol%</td><td>[12]</td></tr>
<tr><td>Hard carbon (Not given/80)|| $Na_{0.67}Mn_{0.6}Ni_{0.2}Cu_{0.1}Co_{0.1}O_2$ (Not given / 70)</td><td>Coin cell 1.9-4.2</td><td>208</td><td>62.6 / 2</td><td>1 mol L^{-1} $NaClO_4$ EC-DMC, 1:1 v/v) 5 vol% FEC</td><td>[27]</td></tr>
<tr><td>$VOPO_4$ (Not given / 80)|| $Na_2Ti_3O_7$ (Not given / 80)</td><td>Pouch cell 2-4.3</td><td>220/1600</td><td>~52 / 0.5</td><td>1 mol L^{-1} $NaClO_4$ in PC:FEC=98:2, vol%</td><td>[2]</td></tr>
<tr><td>FBO@C (1-2 / 80)|| $Na_3Fe_2(PO_4)_2(P_2O_7)$ (5-10 / 80)</td><td>Pouch cell 0-3.5</td><td>175/1680</td><td>44.4 (based on total active material in cathode and anode)/ Not given</td><td>1 mol L^{-1} NaPF6 in PC, EMC, and 5 vol% FEC.</td><td>[3]</td></tr>
<tr><td>Hard carbon (Not given / 80)|| $Na_{0.67}Ni_{0.23}Mg_{0.1}Mn_{0.67}O_2$ (1.4-1.6 / 80)</td><td>Coin cell 2.4-4.25</td><td>249.9/1700</td><td>48 / 0.85</td><td>1 mol L^{-1} $NaClO_4$ in PC:FEC=95:5, vol%</td><td>[1]</td></tr>
<tr><td>Hard carbon (0.7-1.2 / 80)|| $Na_{0.78}Mn_{0.67}Ni_{0.31}Nb_{0.02}O_2$ (2-3 / 80)</td><td>Coin cell 2.3-4.14</td><td>202/7747</td><td>21.8 (based on total active material in cathode and anode)/ 3.68</td><td>1 mol L^{-1} $NaPF_6$ in diglyme</td><td>This work</td></tr>
</table>

Supplementary Table 8. Comparison of low-temperature performance of various cathode materials in coin cell for sodium ion batteries.

	Positive electrode and mass loading (mg cm^{-2}) and active material content (wt.%)	Electrolyte	Anode electrode and mass loading (mg cm^{-2}) and active material content (wt.%)	Operating temperature (°C)	Capacity retention at low-temperature (vs.25 °C)	Ref.
Polyanion materials	NVP-CNTs (3 / 80)	1 mol L^{-1} $NaPF_6$/G2	Bi (1 / 80)	-15	84.3%	28
	$Na_3V_2(PO_4)_2O_2F$ (3.5-4 / 70)	1 mol L^{-1} $NaClO_4$ in EC/PC+5% FEC	Se (1.2-1.5 / 70)	-25	60.7%	29
	NVPF-NTP (1.5-2 / 70)	$NaClO_4$ in EC/PC+5% FEC	Na	-25	76.4%	30
	NVP-C (3 / 80)	Na[FSA]-[C2C1im][FSA] +0.2Na[FSA]	Na	-20	57.1%	31
	NGO@ $Na_3V(PO_3)_3N$ (2.5 / 80)	$NaClO_4$ in EC/PC+5% FEC	Na	-15	75%	32
	$Na_4Fe_3(PO_4)_2(P_2O_7)$ (2.0 / 80)	$NaClO_4$ in EC/PC+5% FEC	Na	-20	87.7%	33
	$Na_4MnCr(PO_4)_3$ (1.2-1.8 / 70)	$NaClO_4$ in PC with 5 % and 10 % FEC	Hard carbon (1.8-2.7 / 70)	-10	79%	34
	NVP@3D porous architectures (Not given / 80)	$NaClO_4$ in EC/PC (1:1) +5% FEC	Na	-20	93%	35
	$Na_3Fe_2(PO_4)_3$ (10 / 70)	$NaClO_4$ in EC/DMC	Hard carbon (10 / 80)	-20	48.3%	36
	Mo-doped $Na_3V_2(PO_4)_3$ (3-3.5 / 70)	$NaClO_4$ in EC/DMC+5 % FEC	Hard carbon (Not given / 70)	-15	95.9%	37
	NVP@C (1 / 80)	$NaClO_4$ in EC/PC	Na	-30	88%	38
Prussian blue material	PB/CNT (Not given / 80)	$NaClO_4$ in EC/PC	Na	-25	85%	39
Layered oxide material	RAHC $Na[Ni_{0.60}Co_{0.05}Mn_{0.35}]O_2$ (Not given / 85)	$NaPF_6$ in EMS (98%) and FEC (2%)	Hard carbon (Not given)	-20	79.7% (vs 30°C)	40
	$Na_{2/3}Ni_{1/3}Mn_{7/12}Fe_{1/12}O_2$ (1 / 75)	$NaClO_4$ in PC+5% FEC	Na	-25	63%	41
	NCM@NTP7 (1.2 / 80)	$NaPF_6$ in a EC/DEC(11 v/v)	Na	-20	80.5%	42
	$Na_{0.67}Ni_{0.2}Co_{0.2}Mn_{0.6}O_2$ (Not given / 80)	$NaPF_6$ in diglyme	Na	-40	78.8%	43
	P2-NaMNNb (2-3 / 80)	$NaPF_6$ in diglyme	Na	-40	98%	This work
			Hard carbon (0.7-1.2 / 80)	-40	95.5%	

Supplementary Notes

Supplementary Note 1. The DFT calculation methods for the substitution of Nb^{5+} in the bulk and surface of P2-NaMNNb.

To better comprehend the mechanism for the Nb substitution on the surface, some models were selected as possible surface planes. The (-103) plane exhibit the lowest formation energy among other calculated planes after structural optimization (Supplementary Figures 7a-c). There are three sites that Nb^{5+} can replace: Mn, Ni and Na site in the bulk phase and surface layer respectively. The calculation results depicted that the formation energy in the bulk of the above three sites are -4.01, -4.67 and -2.29 eV, indicating Ni is the most likely element that replaced by Nb in the bulk phase. However, the formation energy among the three sites of the surface altered obviously according to Supplementary Figure 7d and Table 4, which is -5.59 eV for the Nb substitution in the surface when Nb is in Na site, compared to -4.88 eV and -0.27 eV of Nb in Ni and Mn site, respectively. It is noteworthy that the formation energy is -4.81 eV when all Na replaced by Nb, indicating Nb is prone to occupy Na site and preconstructed the surface due to the trait of its high oxidation state. Furthermore, when Na^{+} replaced by Nb^{5+} on the surface, the charge density around O_{2p} has varied due to the stronger bonding energy of Nb-O than Na-O. The electrostatic interaction between TM and O will alter as the amount of Nb increase, resulting in the transformation process from bulk (*P63/mmc*) to surface (*Fd3m/Fm3m*).

Supplementary Note 2. The analysis of the results of X-ray absorption spectroscopy of Mn and Ni of P2-NaMNNb and P2-NaMN.

To confirm that Nb^{5+} were successfully introduced in the crystal structure of P2-NaMNNb, The X-ray absorption spectroscopy (XAS) were employed to investigate the valance state of Mn and Ni. Supplementary Figure. 12a elucidate the XANES spectra of Mn K-edge of P2-NaMNNb move to lower energy side compared to that of P2-NaMN, with accordance of a slight Mn^{3+} appeared to satisfy charge conservation between anions and cations due to high-valance Nb^{5+} doping in the lattice. The valance of Ni of P2-NaMNNb is almost identical with that of P2-NaMN which close to +2 (Supplementary Figure. 12b). These results demonstrate that Nb^{5+} have been doped into the P2-NaMNNb and have trivial effect on the coordination of TM with similar intensity and profile of the pre-edge peak of P2-NaMN.

Supplementary Note 3. The valance variation analysis of Mn and Ni during charge process of P2-NaMNNb performed by X-ray absorption spectroscopy methods.

Ex situ X-ray absorption spectroscopy (XAS) was performed to verify the valance variation of transition metal ion during electrochemical reaction (Supplementary Figure. 13). It can be seen that the energy shift of K-edge of Mn during electrochemical compensation is not obvious, indicating that Mn ion exhibit minor participation during the electrochemical reaction and remain +4 at the end of charging process (Supplementary Figure. 13a). On the contrary, the K-edge of Ni shifts to a higher energy region upon charge, indicating that Ni^{2+} is oxidized. The energy shift at 4.15 V is ~2.4

eV, which is larger than 2 eV that Ni^{2+}/Ni^{3+} redox alter[16,44,45], suggesting that the Ni ions are responsible for charge compensation during the charge process and the valance state of Ni ions are oxidized from Ni^{2+} to the mixture of Ni^{3+} and Ni^{4+} (Supplementary Figure. 13b). It is estimated that the valance state of Ni ions is +3.2 at the end of charging process, which can deliver the specific capacity of 97.4 mAh g^{-1}, approximately approach the experimental results of 100 mAh g^{-1} at charging process. Therefore, Na^{+} diffusion capability of P2-NaMNNb has been great improved with the dual effects of bulk modulation and surface pre-construction, thus give rise to redox reaction of $Ni^{2+}/Ni^{3+}/Ni^{4+}$ with high voltage plateaus (3.3-4.15 V).

Supplementary Note 4. The valance variation analysis of Mn, Ni and Nb during charge process of P2-NaMNNb and P2-NaMN performed by X-ray photoelectron spectroscopy methods.

The valance states of the elements at different charge/discharge voltages of P2-NaMN and P2-NaMNNb were also investigated by ex situ X-ray photoelectron spectroscopy (XPS). For P2-NaMNNb, the high-resolution spectrum of Ni *2p* reveals that the $2p_{3/2}$ and $2p_{1/2}$ peaks of Ni are 854.2 and 871.6 eV, respectively. When charged to 4.15 V, the spectrum of Ni^{2+} *2p* shifts to the direction of high binding energy (Supplementary Figure. 14b). It shifts to Ni^{3+} and a tiny valence state of Ni^{4+}, indicating oxidation of $Ni^{2+}/Ni^{3+}/Ni^{4+}$[13,44], which means more electrons participate in the redox reaction to contribute more capacity than that of P2-NaMN (Supplementary Figure. 11).

This consequence fits well with the circumstance of CV (Figure. 3a) and charging/discharging curves (Supplementary Figure. 11). After discharged to 2.4 V, the valence of Ni close to +2 with trivial trivalent nickel, this is because the valance of Ni is not completely reduced to +2 at 2.4 V. The XPS Mn *2p* spectrum of P2-NaMNNb (Supplementary Figure. 14c) depicts that co-existence of Mn^{3+} and Mn^{4+} with the ratio of 0.27 : 0.73 owing to the Nb doping. During the charging process, the valence of Mn gradually increases, and only Mn^{4+} exists when reaching 4.15 V. Moreover, the valence of Mn remains +4 after discharged to 2.4 V, indicating that the presence of Mn^{4+} is steady upon cycling. The valence of Nb is +5 during the entire charge and discharge process, which proving that Nb does not participate in charge compensation (Supplementary Figure. 14d).

Supplementary Note 5. The analysis of calculated voltage polarization and IR-drop from GITT data during charge process.

Polarization in GITT curves comprises voltage polarization and ohmic polarization (IR drop), as depicted by the enlarged image inset in Supplementary Figure. 16, which have been reported by previous work.[17,46,47] The voltage polarization of P2-NaMNNb at 3.5 V and 3.8 V at 25 °C should be attributed to the parameter set up during the GITT test. To make the data comparable, we chose the same test step at RT and LT. But the capacity at 25ºC is higher than -40 ºC, no capacity contribution within this voltage range, the voltage polarization will increase once charging stops in this area.

This is also consistent with the drop of diffusion coefficient in 15 h and 30 h in Figure 3c. However, under the same test step, due to the difference of capacity between at 25 °C and -40 °C, the GITT points taken are varied. The above two position of GITT points at 25 °C are obtained without contribution to the capacity, which lead to voltage polarization but negligible IR drop. Besides, we have double checked the result, as shown in Supplementary Figure 16, the IR-drop of P2-NaMNNb at 25 °C is lower than that at -40 °C.

Supplementary Note 6. The analysis of Electron energy loss spectroscopy of P2-NaMNNb powder.

Electron energy loss spectroscopy (EELS) were employed to further figure out the electronic and crystal structure of the Nb-riched surface layer (Supplementary Figure. 27). As depicted in Supplementary Figure. 27b, the Mn-$L_{2,3}$ energy-loss near-edge fine structure (ELNES) shifts towards lower energy gradually from the bulk (regions 1 and 2 in Supplementary Figure. 27a) to the pre-constructed layer (regions 3 and 4 in Supplementary Figure. 27a), indicating that the valence state of Mn in the pre-constructed layer is lower than that in bulk phase (the mixture of Mn^{3+} and Mn^{4+}), which could be attributed to the enrichment of high oxidation state of Nb^{5+} at the surface, reducing Mn^{4+} to Mn^{3+} to satisfy the conservation of charge. Meanwhile, as shown in Supplementary Figure. 27d, the intensities of O K-edge at 530.1 eV decrease with the intensity of pre-edge of O in region 4 being about half of that in region 1, which means

the charge density of TMO_6 of surface layer will transform obviously compared to that of the bulk phase as the variation of pre-edge of O is determined by the charge density and bond energy of TM-O. There is no or small content Na^+ in the pre-constructed layer, so the strong electronegativity of O will localize the 2*p* electrons without the electron holes of Na^+, thereby reducing the TM-O covalency and resulting in less intense pre-edge peaks of O.[48] Besides, the Ni L-edges in the surface is in accordance with that of the bulk phase (Supplementary Figure. 27c), implying that the introduction of Nb^{5+} primarily affects the valence state of Mn, rather than that of Ni.

Supplementary Note 7. The calculation methods for specific capacity, specific energy and specific power of full-cell.

The specific capacity (C_t), specific energy (E) and specific power (P) of full-cell is calculated based on the supplementary equation (1) and (2), as well as (3)[3,4], where E (Wh kg^{-1}) represents the specific energy. t (h) is the total time of the discharge. C_t is the specific capacity of cell. I (mA) is the current of the cell, P (W kg^{-1}) is the specific power of the full-cell, U_a (V) is the average discharge voltage of the cell, which is calculated from the ratio of specific energy (E) to specific capacity (C_t). m_c and m_a (kg) are the mass of P2-NaMNNb in cathode and hard carbon in the anode, respectively.

$$C_t = \frac{I \times t}{m_c + m_a} \quad (1)$$

$$E = C_t \times U_a \quad (2)$$

$$P = \frac{E}{t} \quad (3)$$

Reference

1. Peng, B., Sun, Z., Zhao, L., Li, J. &Zhang, G. Dual-Manipulation on P2-$Na_{0.67}Ni_{0.33}Mn_{0.67}O_2$ Layered Cathode toward Sodium-Ion Full Cell with Record Operating Voltage Beyond 3.5 V. *Energy Storage Mater.* **35,** 620-629 (2021).
2. Li, H., et al. An advanced high-energy sodium ion full battery based on nanostructured $Na_2Ti_3O_7/VOPO_4$ layered materials. *Energy Environ. Sci.* **9,** 3399-3405 (2016).
3. Cao, Y., et al. All-Climate Iron-Based Sodium-Ion Full Cell for Energy Storage. *Adv. Funct. Mater.* **31,** 2102856 (2021).
4. Fang, Y., et al. MXene-Derived Defect-Rich TiO_2@rGO as High-Rate Anodes for Full Na Ion Batteries and Capacitors. *Nano-Micro Letters* **12,** 128 (2020).
5. Liu, X., et al. P2-$Na_{0.67}Al_xMn_{1-x}O_2$: Cost-Effective, Stable and High-Rate Sodium Electrodes by Suppressing Phase Transitions and Enhancing Sodium Cation Mobility. *Angew. Chem. Int. Ed.* **58,** 18086-18095 (2019).
6. Liu, Y., et al. Hierarchical Engineering of Porous P2-$Na_{2/3}Ni_{1/3}Mn_{2/3}O_2$ Nanofibers Assembled by Nanoparticles Enables Superior Sodium-Ion Storage Cathodes. *Adv. Funct. Mater.* **30,** 1907837 (2020).
7. Jo, J. H., et al. Sodium-Ion Batteries: Building Effective Layered Cathode Materials with Long-Term Cycling by Modifying the Surface via Sodium Phosphate. *Adv. Funct. Mater.* **28,** 1705968 (2018).
8. Wang, H., et al. Different Effects of Al Substitution for Mn or Fe on the Structure and Electrochemical Properties of $Na_{0.67}Mn_{0.5}Fe_{0.5}O_2$ as a Sodium Ion Battery Cathode Material. *Inorg. Chem.* **57,** 5249-5257 (2018).
9. Zhu, Y.-F., et al. Manipulating Layered P2@P3 Integrated Spinel Structure Evolution for High-Performance Sodium-Ion Batteries. *Angew. Chem. Int. Ed.* **59,** 9299-9304 (2020).
10. Wang, Q.-C., et al. Tuning Sodium Occupancy Sites in P2-Layered Cathode Material for Enhancing Electrochemical Performance. *Adv. Energy Mater.* **11,** 2003455 (2021).
11. Wang, Y., et al. Ultralow-Strain Zn-Substituted Layered Oxide Cathode with Suppressed P2–O2 Transition for Stable Sodium Ion Storage. *Adv. Funct. Mater.* **30,** 1910327 (2020).
12. Choi, J. U., Jo, J. H., Park, Y. J., Lee, K.-S. &Myung, S.-T. Mn-Rich P′2-$Na_{0.67}[Ni_{0.1}Fe_{0.1}Mn_{0.8}]O_2$ as High-Energy-Density and Long-Life Cathode Material for Sodium-Ion Batteries. *Adv. Energy Mater.* **10,** 2001346 (2020).
13. Liu, Z., et al. Ultralow Volume Change of P2-Type Layered Oxide Cathode for Na-Ion Batteries with Controlled Phase Transition by Regulating Distribution of Na^+. *Angew. Chem. Int. Ed.* **60,** 20960-20969 (2021).
14. Li, X.-L., et al. Stabilizing Transition Metal Vacancy Induced Oxygen Redox by Co^{2+}/Co^{3+} Redox and Sodium-Site Doping for Layered Cathode Materials. *Angew. Chem. Int. Ed.* **60,** 22026-22034 (2021).
15. Yan, Z., et al. A Hydrostable Cathode Material Based on the Layered P2@P3 Composite that Shows Redox Behavior for Copper in High-Rate and Long-Cycling Sodium-Ion Batteries. *Angew. Chem. Int. Ed.* **58,** 1412-1416 (2019).
16. Wang, Q.-C., et al. Tuning P2-Structured Cathode Material by Na-Site Mg Substitution for Na-Ion Batteries. *J. Am. Chem. Soc.* **141,** 840-848 (2019).

17. Xiao, Y., et al. A Stable Layered Oxide Cathode Material for High-Performance Sodium-Ion Battery. *Adv. Energy Mater.* **9,** 1803978 (2019).
18. Liu, K., et al. Insights into the Enhanced Cycle and Rate Performances of the F-Substituted P2-Type Oxide Cathodes for Sodium-Ion Batteries. *Adv. Energy Mater.* **10,** 2000135 (2020).
19. Jin, T., et al. Realizing Complete Solid-Solution Reaction in High Sodium Content P2-Type Cathode for High-Performance Sodium-Ion Batteries. *Angew. Chem. Int. Ed.* **59,** 14511-14516 (2020).
20. Shi, Y., et al. Unlocking the potential of P3 structure for practical Sodium-ion batteries by fabricating zero strain framework for Na^+ intercalation. *Energy Storage Mater.* **37,** 354-362 (2021).
21. Wang, Y., Xiao, R., Hu, Y.-S., Avdeev, M. &Chen, L. P2-$Na_{0.6}[Cr_{0.6}Ti_{0.4}]O_2$ cation-disordered electrode for high-rate symmetric rechargeable sodium-ion batteries. *Nat. Commun* **6,** 6954 (2015).
22. Wang, P.-F., et al. Na^+/vacancy disordering promises high-rate Na-ion batteries. *Science Advances* **4,** eaar6018 (2018).
23. Peng, B., et al. Unusual Site-Selective Doping in Layered Cathode Strengthens Electrostatic Cohesion of Alkali-Metal Layer for Practicable Sodium-Ion Full Cell. *Adv. Mater.* **34,** 2103210 (2022).
24. Zhou, Y.-N., et al. A P2/P3 composite layered cathode for high-performance Na-ion full batteries. *Nano Energy* **55,** 143-150 (2019).
25. Li, X.-L., et al. Whole-Voltage-Range Oxygen Redox in P2-Layered Cathode Materials for Sodium-Ion Batteries. *Adv. Mater.* **33,** 2008194 (2021).
26. Shen, Q., et al. Transition-Metal Vacancy Manufacturing and Sodium-Site Doping Enable a High-Performance Layered Oxide Cathode through Cationic and Anionic Redox Chemistry. *Adv. Funct. Mater.* **31,** 2106923 (2021).
27. Zhao, Q., et al. Tuning oxygen redox chemistry of P2-type manganese-based oxide cathode via dual Cu and Co substitution for sodium-ion batteries. *Energy Storage Mater.* **41,** 581-587 (2021).
28. Wang, C., Du, D., Song, M., Wang, Y. &Li, F. A High-Power $Na_3V_2(PO_4)_3$-Bi Sodium-Ion Full Battery in a Wide Temperature Range. *Adv. Energy Mater.* **9,** 1900022 (2019).
29. Wang, Y.-Y., et al. An Ultralong Lifespan and Low-Temperature Workable Sodium-Ion Full Battery for Stationary Energy Storage. *Adv. Energy Mater.* **8,** 1703252 (2018).
30. Guo, J.-Z., et al. High-Energy/Power and Low-Temperature Cathode for Sodium-Ion Batteries: In Situ XRD Study and Superior Full-Cell Performance. *Adv. Mater.* **29,** 1701968 (2017).
31. Hwang, J., Matsumoto, K. &Hagiwara, R. $Na_3V_2(PO_4)_3$/C Positive Electrodes with High Energy and Power Densities for Sodium Secondary Batteries with Ionic Liquid Electrolytes That Operate across Wide Temperature Ranges. *Advanced Sustainable Systems* **2,** 1700171 (2018).
32. Chen, M., et al. Development and Investigation of a NASICON-Type High-Voltage Cathode Material for High-Power Sodium-Ion Batteries. *Angew. Chem. Int. Ed.* **59,** 2449-2456 (2020).
33. Chen, M., et al. NASICON-type air-stable and all-climate cathode for sodium-ion batteries with low cost and high-power density. *Nat. Commun* **10,** 1480 (2019).
34. Zhang, J., et al. A Novel NASICON-Type $Na_4MnCr(PO_4)_3$ Demonstrating the Energy Density Record of Phosphate Cathodes for Sodium-Ion Batteries. *Adv. Mater.* **32,** 1906348 (2020).
35. Rui, X., et al. A Low-Temperature Sodium-Ion Full Battery: Superb Kinetics and Cycling Stability. *Adv. Funct. Mater.* **31,** 2009458 (2021).

36. Cao, Y., et al. Highly Stable $Na_3Fe_2(PO_4)_3$@Hard Carbon Sodium-Ion Full Cell for Low-Cost Energy Storage. *ACS Sustainable Chemistry & Engineering* **8,** 1380-1387 (2020).
37. Liang, L., et al. Construction and Operating Mechanism of High-Rate Mo-Doped $Na_3V_2(PO_4)_3$@C Nanowires toward Practicable Wide-Temperature-Tolerance Na-Ion and Hybrid Li/Na-Ion Batteries. *Adv. Energy Mater.* **11,** 2100287 (2021).
38. Liu, T., et al. All-climate sodium ion batteries based on the NASICON electrode materials. *Nano Energy* **30,** 756-761 (2016).
39. You, Y., et al. Subzero-Temperature Cathode for a Sodium-Ion Battery. *Adv. Mater.* **28,** 7243-7248 (2016).
40. Hwang, J.-Y., et al. Radially aligned hierarchical columnar structure as a cathode material for high energy density sodium-ion batteries. *Nat. Commun* **6,** 6865 (2015).
41. Yang, Q., et al. Advanced P2-$Na_{2/3}Ni_{1/3}Mn_{7/12}Fe_{1/12}O_2$ Cathode Material with Suppressed P2–O2 Phase Transition toward High-Performance Sodium-Ion Battery. *Acs Appl. Mater. Inter* **10,** 34272-34282 (2018).
42. Li, Y., et al. Construction nasicon-type $NaTi_2(PO_4)_3$ nanoshell on the surface of P2-type $Na_{0.67}Co_{0.2}Mn_{0.8}O_2$ cathode for superior room/low-temperature sodium storage. *Chem. Eng. J.* **402,** 126181 (2020).
43. Wang, X., et al. Rational design of $Na_{0.67}Ni_{0.2}Co_{0.2}Mn_{0.6}O_2$ microsphere cathode material for stable and low temperature sodium ion storage. *Chem. Eng. J.* **428,** 130990 (2022).
44. Ma, C., et al. Exploring Oxygen Activity in the High Energy P2-Type Na0.78Ni0.23Mn0.69O2 Cathode Material for Na-Ion Batteries. *J. Am. Chem. Soc.* **139,** 4835-4845 (2017).
45. Yabuuchi, N., Yoshii, K., Myung, S.-T., Nakai, I. &Komaba, S. Detailed Studies of a High-Capacity Electrode Material for Rechargeable Batteries, $Li_2MnO_3-LiCo_{1/3}Ni_{1/3}Mn_{1/3}O_2$. *J. Am. Chem. Soc.* **133,** 4404-4419 (2011).
46. Shen, Z., Cao, L., Rahn, C. D. &Wang, C.-Y. Least Squares Galvanostatic Intermittent Titration Technique (LS-GITT) for Accurate Solid Phase Diffusivity Measurement. *J. Electrochem. Soc.* **160,** A1842-A1846 (2013).
47. Weppner, W. &Huggins, R. A. Determination of the Kinetic Parameters of Mixed-Conducting Electrodes and Application to the System Li3Sb. *J. Electrochem. Soc.* **124,** 1569-1578 (1977).
48. Liu, S., et al. Li–Ti Cation Mixing Enhanced Structural and Performance Stability of Li-Rich Layered Oxide. *Adv. Energy Mater.* **9,** 1901530 (2019).
